# Genome-wide association mapping of partial resistance to *Phytophthora sojae* in soybean plant introductions from the Republic of Korea

**DOI:** 10.1186/s12864-016-2918-5

**Published:** 2016-08-11

**Authors:** Rhiannon Schneider, William Rolling, Qijian Song, Perry Cregan, Anne E. Dorrance, Leah K. McHale

**Affiliations:** 1 Department of Horticulture and Crop Science, The Ohio State University, Columbus, OH 43210 USA; 2 Present Address: Pioneer Hi-Bred International Inc., Napoleon, OH 43545 USA; 3 Center for Applied Plant Sciences, The Ohio State University, Columbus, OH 43210 USA; 4 US Department of Agriculture, Soybean Genomics and Improvement Laboratory, Agricultural Research Service, Beltsville, MD 20705 USA; 5 Department of Plant Pathology, The Ohio State University, Wooster, OH 44691 USA

**Keywords:** *Glycine max*, GWAS, Haplotype, Linkage disequilibrium, Partial resistance, *Phytophthora sojae*, Single nucleotide polymorphism

## Abstract

**Background:**

Phytophthora root and stem rot is one of the most yield-limiting diseases of soybean [*Glycine max* (L.) Merr], caused by the oomycete *Phytophthora sojae*. Partial resistance is controlled by several genes and, compared to single gene (*Rps* gene) resistance to *P. sojae,* places less selection pressure on *P. sojae* populations. Thus, partial resistance provides a more durable resistance against the pathogen. In previous work, plant introductions (PIs) originating from the Republic of Korea (S. Korea) have shown to be excellent sources for high levels of partial resistance against *P. sojae*.

**Results:**

Resistance to two highly virulent *P. sojae* isolates was assessed in 1395 PIs from S. Korea via a greenhouse layer test. Lines exhibiting possible *Rps* gene immunity or rot due to other pathogens were removed and the remaining 800 lines were used to identify regions of quantitative resistance using genome-wide association mapping. Sixteen SNP markers on chromosomes 3, 13 and 19 were significantly associated with partial resistance to *P. sojae* and were grouped into seven quantitative trait loci (QTL) by linkage disequilibrium blocks. Two QTL on chromosome 3 and three QTL on chromosome 19 represent possible novel loci for partial resistance to *P. sojae*. While candidate genes at QTL varied in their predicted functions, the coincidence of QTLs 3-2 and 13-1 on chromosomes 3 and 13, respectively, with *Rps* genes and resistance gene analogs provided support for the hypothesized mechanism of partial resistance involving weak *R*-genes.

**Conclusions:**

QTL contributing to partial resistance towards *P. sojae* in soybean germplasm originating from S. Korea were identified. The QTL identified in this study coincide with previously reported QTL, *Rps* genes, as well as novel loci for partial resistance. Molecular markers associated with these QTL can be used in the marker-assisted introgression of these alleles into elite cultivars. Annotations of genes within QTL allow hypotheses on the possible mechanisms of partial resistance to *P. sojae*.

**Electronic supplementary material:**

The online version of this article (doi:10.1186/s12864-016-2918-5) contains supplementary material, which is available to authorized users.

## Background

Phytophthora root and stem rot was the second most yield-limiting disease of soybean [*Glycine max* (L.) Merr] between 1996 and 2009 [1, 2]. This disease, caused by the soil-borne oomycete pathogen *Phytophthora sojae* [3], is prevalent when soil conditions become saturated [4], allowing the asexual, motile zoospores to chemotactically travel to soybean roots [5, 6]. Upon infection, *P. sojae* will produce haustoria and acquire nutrients in a hemi-biotrophic manner [7]. Susceptible plants will develop lesions, experience wilting and chlorosis, and, in severe cases, plant death [4].

Genetic resistance is an effective strategy to manage *P. sojae* in regions with high levels of inoculum and favorable environments [8]. Soybean breeding programs have primarily utilized single, dominant *Rps-*mediated resistance in which recognition of *P. sojae* effector proteins initiate effector-triggered immunity, resulting in complete resistance. To date, 21 *Rps* genes or alleles have been identified and mapped to five chromosomes [9–18]. Though potentially highly effective, *Rps-*mediated resistance is race-specific and effectiveness of a given *Rps* gene is dependent on the population of *P. sojae* present. Additionally, deployment of *Rps* genes places high selection pressures on the *P. sojae* populations causing the population to adapt and potentially gain virulence such that the *Rps* gene is no longer effective. Widespread deployment of *Rps* genes in soybean cultivars has resulted in the evolution of highly diverse *P. sojae* populations [19] with more than 200 physiological races (55 described) identified in the US [20–24]. Pathogen diversity and adaptation limits the efficacy of an *Rps* gene to eight to twenty years [8, 25] thus, breeders cannot rely solely on *Rps* genes.

In contrast to *Rps-*mediated resistance, partial resistance is a quantitative trait, controlled by multiple genes at numerous loci, each contributing a small effect or a few loci contributing a moderate effect [26–28]. Partial resistance to *P. sojae* has been shown to be effective against numerous pathotypes of *P. sojae* [29, 30]*.* Unlike *Rps*-mediated resistance, partial resistance to *P. sojae* is incomplete, and allows some pathogen growth and reproduction [30]. This is believed to place minimal selection pressure on the *P. sojae* populations exposed to cultivars possessing partial resistance. For this reason partial resistance is predicted to be more durable with examples such as powdery mildew management in winter wheat indicating effectiveness on the scale of decades [31, 32]. Multiple mechanisms for partial resistance have been broadly hypothesized [26–28]. Mechanistic studies specific to this pathosystem have provided evidence for the involvement of *R*-genes [33], components of defense signal transduction pathways [33–35] and genes regulating plant physiology or morphology [33, 35], including suberin deposition in the root [36, 37] in partial resistance to *P. sojae*.

Improved levels of partial resistance against *P. sojae* in US soybean cultivars can be achieved through the introgression of novel alleles. Over 1000 soybean plant introductions (PIs) were previously evaluated as potential novel sources of resistance to *P. sojae* and those originating from The Republic of Korea (S. Korea) were associated with high levels of partial resistance [38]. Therefore, it was proposed that high genetic diversity for *P. sojae* resistance may exist in PIs from S. Korea as a result of the potential co-evolution between soybean and *P. sojae* that may have occurred in this region [38].

Identification of QTL for partial resistance against *P. sojae* in soybean has been limited to the cultivar Conrad [39–44], southern germplasm V71-370 [45], and eight accessions originating from Asia [46–50]; including four PIs originating from S. Korea [47–50]. Among the PIs from S. Korea, QTL for partial resistance to *P. sojae* were identified on all chromosomes (Chrs), except 5, 6, 11, 17 and 19, with between two and eight QTL identified in each population and most QTL contributing less than 10 % of the phenotypic variation (PVE) [47–50]. Interestingly, in a recombinant inbred line (RIL) population derived from a cross between PI 427105B and the susceptible breeding line OX20-8, a QTL with the largest PVE (up to 45 %) was identified on Chr 18 [47].

Given the success in identifying and diversity of QTL contributing to partial resistance from PIs, it is pertinent to further evaluate a broader array of PIs for partial resistance and to identify the common alleles that may be contributing to resistance within this germplasm source. In this study 1395 PIs originating from S. Korea were evaluated for partial resistance to *P. sojae*. A genome-wide association (GWA) analysis was performed using high-density genetic markers from the Soy50KSNPChip [51]. In this study, the extent of root rot, root weight, shoot weight and plant height from inoculated plants were combined with genotypic data to identify 16 markers significantly associated with these traits across three chromosomes. Associated markers were grouped into seven QTL according to linkage disequilibrium and candidate genes were identified within these regions.

## Results and discussion

### Phenotypic data

**Fig. 1:**
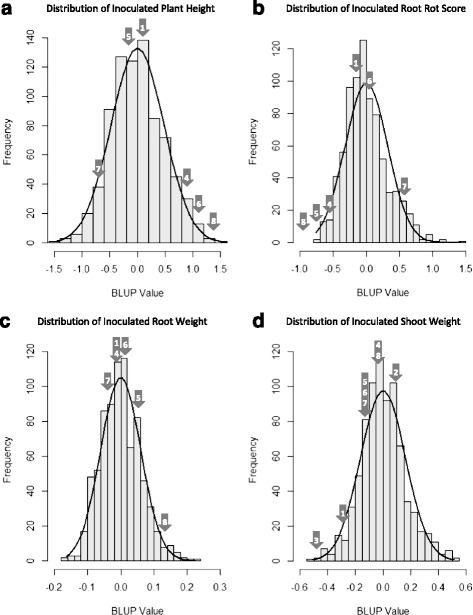
Distribution of BLUP values for inoculated traits. Histograms are depicted for inoculated plant height (**a**), inoculated root rot score (**b**), inoculated root weight (**c**) and inoculated shoot weight (D). Numbered arrows in histograms indicate BLUE values of checks (1 = Conrad, 2 = L83-570, 3 = OX-20, 4 = PI398841, 5 = PI407861A, 6 = Resnik, 7 = Sloan, and 8 = Williams 79)

Over 1300 PIs originating from S. Korea were evaluated for their response to two virulent isolates of *P. sojae*, *C2S1* (*vir 1a*, *1b*, *1c, 1d*, *1k*, *2*, *3a*, *3b*, *3c*, *4*, *5*, *6*, *7* and *8*) and *OH12108_6.3* (*OH121*; *vir 1a*, *1b*, *1c*, *1d*, *1k*, *2*, *3a*, *3c*, *4*, *5*, *6*, *7* and *8*), using the greenhouse-based layer test assay. Phenotypic data were collected for inoculated root rot score (IRRS; 1, no rot, to 9, completely rotted) as well as for root weight (RW), shoot weight (SW) and plant height (PH) from inoculated (I) and non-inoculated (N) assays. A total of 266 PIs were excluded from the final data set due to poor germination or missing data. There was no observable root rot in 306 PIs (IRRS ≤ 1.5) following inoculation with one or both isolates of *P. sojae* and these were excluded from analysis in order to eliminate any possible *Rps*-mediated resistance response. In addition, 23 PIs possessed a mean non-inoculated root rot score > to 1.5 and were removed from the analysis to exclude any effects from potential seed-borne pathogens. The remaining 800 PIs had a mean IRRS of 3.5 which ranged from 1.6 to 8.0. Best linear unbiased predictor (BLUP) values were calculated for each PI, representing the PI’s genotypic value for each trait. Similarly, best linear unbiased estimator (BLUE) values were calculated for each check class, which was treated as a fixed effect. In comparison to check cultivars, the PIs generally exhibited high levels of partial resistance. Twenty-seven PIs had lower IRRS BLUP values than the mean IRRS BLUE values for highly resistant checks (Conrad, PI407861A and PI398841); whereas no PIs had IRRS BLUP values that exceeded the mean BLUE values for either the moderately susceptible check (Sloan) or the highly susceptible check (OX20-8). IRW, ISW, IPH showed normal distributions and IRRS had a slightly positive skew (Fig. 1).

All four inoculated traits were significantly correlated with each other, where IRW, ISW and IPH were positively correlated, and IRRS, in which a lower value indicates greater resistance, was negatively correlated to IRW, ISW and IPH (Table 1). There was significant genetic variance for the four traits in both inoculated and non-inoculated treatments (Table 2). The non-inoculated traits, NRW, NSW and NPH were all highly heritable, greater than 0.720. The heritability for the four inoculated traits ranged from moderately low at 0.334 for IRW to moderately high at 0.605 for ISW (Table 2). In an analysis of assays with each isolate of *P. sojae* separately, resistance towards *C2S1* had lower heritability and lower disease incidence compared to *OH121*, where the mean of the raw IRRS for *C2S1* and *OH121* was 2.85 and 4.13, respectively. While the disparity in heritability between isolates are similar to observations in previous reports [48, 52] and can likely be attributed to the reduced disease development from the *C2S1* isolate, it emphasizes the need to carry out assays for partial resistance with multiple isolates. Although the *C2S1* isolate was virulent in the hypocotyl test and moderately aggressive in a tray test (Additional file 1: Table S1), the aggressiveness of *P. sojae* isolates can vary depending upon which component of partial resistance is measured in the phenotypic disease assay [30, 52].

**Table 1:** Pearson’s correlation coefficients (top right) and *p*-values (bottom left) for inoculated root rot score (IRRS), inoculated root weight (IRW), inoculated shoot weigh (ISW) and inoculated plant height (IPH)

	IRRS	IRW	ISW	IPH
IRRS		−0.67	−0.62	−0.5
IRW	<0.0001		0.78	0.51
ISW	<0.0001	<0.0001		0.58
IPH	<0.0001	<0.0001	<0.0001

**Table 2:** Genetic variance (σ_g_^2^), variance of isolate (σ_i_^2^), genotype by isolate variance (σ_gi_^2^), variance of error (σ_e_^2^) and heritability (*H* ^*2*^) of each trait

Isolate	Trait^a^	σ_g_^2^	σ_i_^2^	σ_gi_^2^	σ_ε_^2^	*H* ^*2*^
*C2S1* and *OH121* ^b^	IRRS	0.29***	0.83	0.22***	1.52***	0.23
	IRW	0.01***	0.00	0.01**	0.08***	0.20
	ISW	0.05***	0.00	0.01**	0.10***	0.78
	IPH	0.65***	0.00	0.82***	2.75***	0.71
*OH121*	IRRS	0.75***	NA^c^	NA	1.51***	0.50
	IRW	0.03***	NA	NA	0.06***	0.44
	ISW	0.07***	NA	NA	0.07***	0.67
	IPH	1.43***	NA	NA	3.75***	0.43
*C2S1*	IRRS	0.24**	NA	NA	1.55***	0.24
	IRW	0.01**	NA	NA	0.09***	0.23
	ISW	0.05***	NA	NA	0.12***	0.43
	IPH	1.45***	NA	NA	1.88***	0.61
NA	NRW	0.04***	NA	NA	0.06***	0.72
	NSW	0.09***	NA	NA	0.10***	0.78
	NPH	1.15***	NA	NA	1.90***	0.71

Asterisks indicate the level of statistical significance: * *P* ≤ 0.05; ** *P* ≤ 0.01; *** *P* ≤ 0.001

^a^ *IRRS* inoculated root rot score, *IRW* inoculated root weight, *ISW* inoculated shoot weight, *IPH* inoculated plant height, *NRW* non-inoculated root weight, *NSW* non-inoculated shoot weight, *NPH* non-inoculated plant height

^b^ *OH121*: *OH121086.3*

^c^Not applicable

### Population structure and linkage disequilibrium

**Fig. 2:**
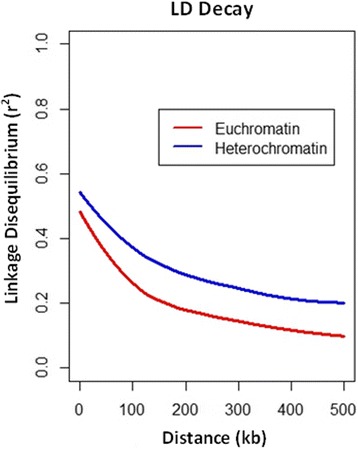
Linkage disequilibrium (*r* ^2^) as a function of physical distance (kb)

Analysis of population structure among the 800 PIs that were included in the GWA analysis using ADMIXTURE [53] indicated a continued decline in cross-validation (CV) error as values of *K* were tested from 1 to 22 (Additional file 1: Figure S1A). However, subpopulations defined at a local minimum of *K* = 3 (Additional file 1: Figure S1B) corresponded to differentiation of PIs by principle component analysis and was determined to be the most likely number of subpopulations (Additional file 1: Figure S1C). A total of 19,303 polymorphic markers were used to carry out GWA mapping in this population. Markers were at an average genome-wide density of one marker every 50.5 kb or, more specifically, at a density of one marker every 29.9 and 124.4 kb in euchromatic and heterochromatic regions, respectively. LD decayed to half of its initial value at approximately 7.32 and 17.7 kb in the euchromatin and heterochromatic regions, respectively. Due to LD among markers, the 19,303 markers could be condensed into 12,313 effective markers, or an average of one effective marker approximately every 80 kb. Linkage disequilibrium (LD) decayed to an *r*^*2*^ of 0.2 at approximately 172.3 and 556.6 kb in euchromatin and heterochromatic regions, respectively (Fig. 2), indicating that, on average, marker density is sufficient to capture the majority of the genome. This effective number of markers (M_eff_) was considered the number of independent tests and employed in a correction for multiple testing in which marker trait associations were considered significant when the *P-*value was less than α/M_eff_, 4.06 × 10^−6^ [54].

### GWA analysis

**Fig. 3:**
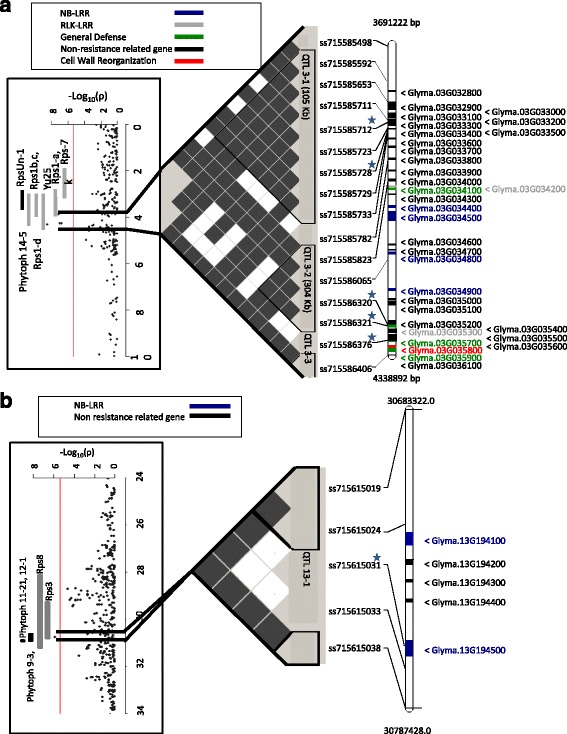
QTLs identified for root rot score on chromosome 3 (**a**) and for inoculated root weight on chromosome 13 (**b**). The far left image is a Manhattan plot indicating the level of marker association with either root rot score or inoculated root weight. The central image contains a visualization of linkage disequilibrium (white is an r^2^ value of zero, black is an r^2^ value of 1). Significant haplotype blocks are outlined in black. The far right image is a physical map of markers (stars indicate markers significantly associated with inoculated root rot score) and candidate genes with text color indicate the general annotation categories related to resistance

**Fig. 4:**
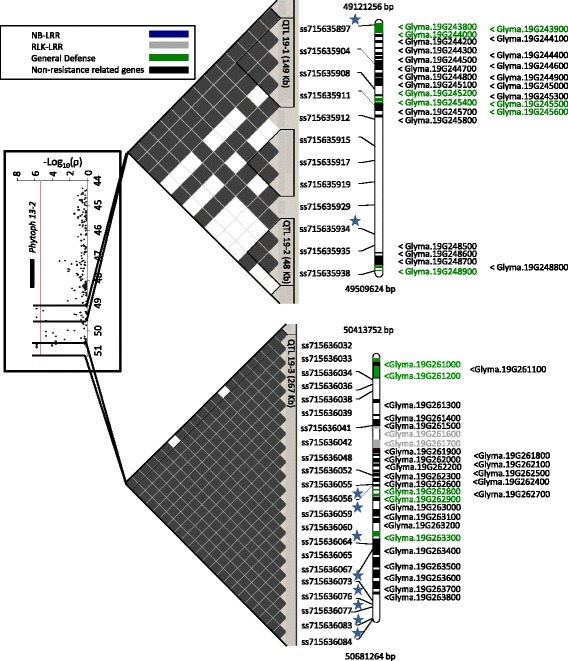
QTLs identified on chromosome 19 for root rot score. The far left image is a Manhattan plot indicating the level of marker association with either root rot score or inoculated root weight. The central image contains a visualization of linkage disequilibrium (white is an r^2^ value of zero, black is an r^2^ value of 1). Significant haplotype blocks are outlined in black. The far right image is a physical map of markers (stars indicate markers significantly associated with inoculated root rot score) and candidate genes with text color indicate the general annotation categories related to resistance

GWA analysis of partial resistance to *P. sojae* has been limited to two studies, one which used 174 soybean accessions from the mini core collection of cultivated soybean from China with 495 SSR loci [55] and recently a second study used 472 accessions from a Chinese breeding program with nearly 60,000 SNPs [56]. As a result, little information is available regarding the genetic distribution of alleles for partial resistance in a breadth of germplasm. The present study utilized nearly 800 PIs and employed the largest population of any previously GWA analyses performed for disease resistance in soybean [55–60]. A total of 16 significant marker-trait associations were identified for IRRS and IRW (Table 2). These mapped to genomic regions on Chrs 3, 13 and 19 (Figs. 3 and 4). While the large number of accessions assayed and limited availability of seed prohibited an evaluation of a potential *Rps* gene mediated response for each isolate and accession combination, the methods used in this study applied several approaches to avoid *Rps*-mediated responses. These approaches included the selection of complex isolates, the removal of accessions exhibiting limited root rot in inoculations with either C2S1 or OH121, as well as the inclusion of a genotype-by-isolate interaction term in the model applied. In addition, hypocotyl assays carried out on 94 randomly selected accessions included in the GWA analysis showed a virulent reaction of C2S1, indicating a lack of *Rps*-mediated resistance within these accessions (Additional file 1: Table S2). Within this group of accessions there is one or more individuals possessing the resistance allele for 11 out of the 16 significant markers. Due to these approaches implemented in this study, it is expected that the significant marker-trait associations represent partial resistance loci. However, it cannot be ruled out that *Rps*-mediated resistance has been observed in a quantitaive manner due to incomplete resistance as observed in the root gene *Rps2* [30] or errors associated with scoring of root rot.

Genomic regions on Chrs 3 and 19 were identified with significant marker-trait associations for IRRS. Five SNPs on Chr 3 (ss715585728, ss715585712, ss715586321, ss715586320, ss715586376) were located within a 0.4 Mb region (3.9–4.3 Mb) (Fig. 3a). ss715586376 was the most significantly associated SNP with a *P*-value of 3.27 × 10^−9^ (Table 3). Ten SNPs on Chr 19 (ss715635897, ss715635934, ss715636056, ss715636059, ss715636064, ss715636073, ss715636076, ss715636077, ss715636083, ss715636084) were significantly associated with IRRS and were located within a 1.6 Mb region (49.4–50.7 Mb) (Fig. 4), with all ten SNPs possessing a *P*-value of 1.82 × 10^−6^ (Table 3).

**Table 3:** Significant marker-trait associations with identified genomic regions and chromosome location for each inoculated trait and model

Trait	Marker	Chr.^a^	Chr. position (bp^b^)	QTL	Minor allele frequency	Additive effect	PVE^c^	*p*-value
Inoculated Root Rot Score	ss715585712	3	3852888	3-1	0.18	0.096	3.162	9.14E-08
	ss715585728	3	3865730	3-1	0.18	0.0967	3.212	7.26E-08
	ss715586320	3	4276534	3-2	0.21	0.0922	3.018	1.77E-07
	ss715586321	3	4277380	3-2	0.21	0.0936	3.133	1.04E-07
	ss715586376	3	4315512	3-3	0.2	0.1039	3.895	3.27E-09
Inoculated Root weight	ss715615031	13	30766058	13-1	0.42	−0.0695	2.468	1.44E-06
Inoculated Root Rot Score	ss715635897	19	49121258	19-1	0.01	−0.4622	2.513	1.82E-06
	ss715635934	19	49461582	19-2	0.01	−0.4622	2.513	1.82E-06
	ss715636056	19	50544363	19-3	0.01	−0.4622	2.513	1.82E-06
	ss715636059	19	50555433	19-3	0.01	−0.4622	2.513	1.82E-06
	ss715636064	19	50604933	19-3	0.01	−0.4622	2.513	1.82E-06
	ss715636073	19	50663466	19-3	0.01	−0.4622	2.513	1.82E-06
	ss715636076	19	50666563	19-3	0.01	−0.4622	2.513	1.82E-06
	ss715636077	19	50668662	19-3	0.01	−0.4622	2.513	1.82E-06
	ss715636083	19	50679714	19-3	0.01	−0.4622	2.513	1.82E-06
	ss715636084	19	50681263	19-3	0.01	−0.4622	2.513	1.82E-06

^a^ *Chr* chromosome

^b^ *bp* basepair position in the Glyma.Wm82.a2 assembly

^c^ *PVE* percent variance explained

A single SNP (ss715615031) at 30.7 Mb on Chr 13 was significantly associated with IRW (*P*-value = 1.44 × 10^−6^) (Table 3; Fig. 3b). While no significant marker-trait associations were identified for ISW or IPH, for IPH several near significant markers at ~3.9 Mb on Chr 3 and ~30 Mb on Chr 13 were noted (Additional file 1: Figure S2). Near significance of ss715615031, the significant marker for IRW, on Chr 13 (*P*-value = 4.83 × 10^−6^) and near significance of ss715585728, one of the significant markers for IRRS, on Chr 3 (*P*-value = 1.11 × 10^−5^) were observed for IPH.

No significant marker-trait associations were found in the NRW or NPH (Additional file 1: Figure S3). Five genomic regions were identified with significant marker associations for NSW on Chr 2 (3.4–4.6 Mb), 3 (5.2–5.6 Mb), 4 (6.1–6.6 Mb), 17 (8.5 Mb), and 18 (51.7–53.0 Mb). However, none of the significant markers for NSW were coincident with the markers identified for the inoculated traits, IRRS and IRW.

### Grouping of significantly associated SNPs into QTL

A QTL was defined as a haplotype block possessing marker(s) identified as significantly associated with a trait. Based on this criterion, the 16 markers significantly associated with IRRS or IRW were grouped into seven QTL ranging in size from 176 to 48 kb. The extensive historical recombination present in a population of PIs can lead to the identification of relatively narrow QTL in GWA analysis in comparison to mapping conducted in bi-parental populations. In previous mapping studies conducted with RIL and NAM populations, identified QTL spanned an average of 6132.6 kb, with the largest QTL encompassing 940 genes [39–50, 52]. In contrast, the largest QTL in this study is 304 kb in length and contains 13 genes.

### Candidate genes and coincident traits for QTL 3-1, 3-2 and 3-3

The five significant marker-trait associations on Chr 3 were grouped into two haplotype blocks, each possessing two significant marker-IRRS associations. A fifth marker which was significantly associated with IRRS, was not in significant LD with neighboring markers. These regions are referred to as QTL 3-1, 3-2 and 3-3, respectively (Fig. 3a). QTL 3-1 is coincident with a previously identified QTL (*Phytoph 14-5*; www.soybase.org [61]) for lesion length in a tray test of partial resistance to *P. sojae* where the resistance allele was from a PI originating from S. Korea [52]. A QTL associated with resistance to the necrotrophic pathogen, *Sclerotinia sclerotiorum,* was also in close proximity to QTL 3-1 [59, 62]. Additionally, QTL 3-1 partially overlaps with *Rps1a, b, c, d* and *k*; *RpsYu25*; and *RpsUn1* and is also nearby the putative position of *Rps7* [9, 10, 12, 15, 63] (Fig. 3a). QTL 3-1 contains eight predicted genes in a 105.5 kb region (Glyma.Wm82.a2.v1, accessed Phytozome v10; Additional file 2: Table S3). Interestingly, while QTL 3-1 partially overlaps with a number of *Rps* genes, none of the eight predicted genes from the Williams 82 reference sequence within this QTL were conventionally considered to be related to defense or resistance. Therefore, QTL 3-1 may be conferred by non-canonical *R*-genes or, more likely, is located upstream of the *Rps* genes and associated with a novel mechanism for partial resistance.

The second QTL (3-2) is 238.1 kb downstream of QTL 3-1 and is coincident with the mapped positions of *Rps1d* and *RpsUn1* [10, 15]*.* This region does not coincide with any QTL for partial resistance to *P. sojae* (Fig. 3a). Thus, QTL 3-2 may represent a novel QTL for partial resistance to *P. sojae*. QTL 3-2 encompasses 304 kb and 13 predicted genes (Additional file 2: Table S3), seven of which have putative functions relating to disease resistance or defense. Four of these genes (Glyma.03G034400, Glyma.03G034500, Glyma.03G034800 and Glyma.03G034900) putatively encode a nucleotide binding (NB) domain characteristic of *R*-genes [64]. In addition, there are two leucine-rich repeat receptor-like kinase (LRR-RLK)encoding genes in this region (Glyma.03G034200 and Glyma.03G035300), a class of genes involved in basal defense and plant developmental responses [65–68]. Finally, Glyma.03G034100 is homologous to a Sec61 protein transporter-encoding gene, which is involved in activation of systemic-acquired resistance in Arabidopsis [69].

Marker ss715586376 is located 238.1 kb downstream of QTL 3-2 and was significantly associated with IRRS. Marker ss715586376 was not in LD with nearby markers, thus, the QTL 3-3 region was defined by the two flanking markers, ss715586321 and ss715586406 (Fig. 3a). There are a total of seven genes between these markers (Additional file 2: Table S3), of which three have possible roles in defense. Glyma.03G035700 putatively encodes an abscisic acid responsive stress related protein [70]. Glyma.03G035800 encodes a putative Alpha-expansin involved in cell wall extension and growth [71, 72]. Glyma.03G035900 is a CAD1 encoding gene with Mac/perforin domains. The CAD1 encoding genes have been shown to be involved in plant defense in Arabidopsis by activating the salicylic acid pathway causing a hypersensitive response [73].

### Candidate genes and coincident traits for QTL 13-1

Located on Chr 13, ss71565031 was significantly associated with IRW (Fig. 3b). Similar to QTL 3-3, ss71565031 was not located in a haplotype block. Therefore, QTL 13-1 region was defined by the two flanking markers, ss715615924 and ss715615033 and spans 49.2 kb. QTL 13-1 is coincident with *Rps8* and *Rps3* [9, 17]*,* three previously identified QTL for partial resistance against *P. sojae* (*Phytoph 11-21*, *9*-*3*, *12*-*1* [61]) [43, 48, 52], a QTL for flood tolerance [48], and QTL for resistance against *S. sclerotiorum* [62]*.* The region contains a total of five predicted genes (Additional file 2: Table S3), of which two (Glyma.13G194100 and Glyma.13G194500) are NB-LRR encoding genes.

### Candidate genes and coincident traits for QTL 19-1, 19-2 and 19-3

A total of ten significant markers for the IRRS were located on Chr 19. The significant markers were grouped into three QTL based on haplotype blocks, QTL 19-1, QTL 19-2 (Fig. 4) and QTL 19-3 (Fig. 4). No previously identified QTL associated with resistance to *P. sojae* or other pests or pathogens were coincident with the three QTL on Chr 19. However, marker ss715635897 from QTL 19-1 is located approximately 1.1 Mb from a previously identified QTL (*Phytoph13-2* [61]) for partial resistance to *P. sojae* through a tray test disease assay [52].

QTL 19-1 spans 149 kb and contains a total of 21 predicted genes (Fig. 4; Additional file 2: Table S3), of which seven are putative defense related genes. The defense genes in the region include Glyma.19G245400, Glyma.19G245500 and Glyma.19G245600, which encode putative PR4-related chitin-binding proteins [74]. Glyma.19G243800 encodes a putative ribose 5-phosphate isomerase. Homologs in *Arabidopsis* have been shown to function in cellulose synthase [75], which has been shown to be involved in the regulation of jasmonic acid and ethylene [76] with mutants displaying enhanced resistance to bacterial and fungal pathogens [77]. Other genes in the region include Glyma.19G244000 that encodes a putative MATE efflux protein potentially involved in defense signaling pathways or in transporting toxic compounds from infected cells [78, 79] as well as Glyma.19G245200 that encodes a putative auxin responsive gene [70]. Finally, Glyma.19G244400 encodes a putative ammonium transporter, homologs of which have been implicated in interactions with root endophytes [80–82] and can act as a negative regulator of basal defense responses in *Arabidopsis* [83]*.*

QTL 19-2 is 48 kb in length and located 218 kb downstream of QTL 19-1 (Fig. 4). This QTL encompassed five predicted genes (Additional file 2: Table S3) including Glyma.19G248900 that encodes a putative ethylene/JA responsive transcription factor [70].

The third QTL identified on Chr 19 (QTL 19-3) is 267 kb in length and is located 904 kb downstream of QTL 19-2 (Fig. 4). It encompasses a total of 29 predicted genes (Additional file 2: Table S3), six of which have annotations representative of possible defense function. These genes include Glyma.19G261000 and Glyma.19G261700 that putatively encode LRR-RLKs, Glyma.19G261200 that is a dicer family protein putatively involved in the control of gene silencing [84], Glyma.19G262800 and Glyma.19G262900 that putatively encode GDSL esterase/lipases which can be associated with the ethylene/JA responsive defense pathways [85–87], and Glyma.19G263300 that encodes a putative ethylene/JA responsive lipoxygenase [88].

### Inferences about mechanisms of partial resistance

It has been hypothesized that partial disease resistance can be controlled through developmental or morphological mechanisms, basal defense genes, production of antimicrobials or detoxification of phytotoxins (chemical warfare), components of defense signal transduction pathways, weak *R*-gene responses or other unknown mechanisms [27]. While functional gene analysis is required to identify mechanisms, the co-localization of annotated genes and QTLs can theoretically provide evidence in support of particular mechanisms. In this study, co-localization of annotated genes and QTLs provides varying levels of support for each of the aforementioned hypotheses.

Mechanisms of partial resistance associated with developmental or morphological mechanisms are difficult to assess through annotations of co-localized genes because there are a limited number of clearly defined pathways for these complex traits. However, QTLs 3-3 and 19-1 contain candidate genes putatively involved in morphology and development, including cell growth and cellulose production. Evidence for basal defense was found through co-localization of QTLs 3-2 and 19-3 with LRR-RLK-encoding genes. However, while LRR-RLKs are known to be involved in recognition of microbe associated molecular patterns leading to basal defense responses [65–67], LRR-RLK-encoding genes have also been implicated in a range of functions, including plant development [68]. Transport of toxic compounds is a potential function of a MATE efflux-encoding gene within QTL 19-1, providing a candidate for the chemical warfare hypothesis. QTLs 3-2, 3-3, 19-1 and 19-3 encompass genes involved in defense signal transduction, with putative functions ranging from negative regulators of basal defense to the control of gene silencing. Among the largest class of genes within these QTL are NB-LRR-encoding genes [64]. In support of the weak *R*-gene hypothesis, QTLs 3-2 and 13-1 are coincident with six NB-LRR-encoding genes and overlap with estimated positions of *Rps* genes [9, 10, 12, 15, 63]. Finally, QTL 3-1 does not contain any genes normally associated with defense or development pertinent to *P. sojae* resistance and therefore supports the idea that quantitative defense can be conferred by a yet unknown class of genes [27].

Consistent with previous studies [33], the QTL encompass genes functioning in a wide range of processes, potentially indicating that a number of different mechanisms contribute to quantitative defense, making this germplasm a welcome resource to diversify resistance genes. The current study along with previous QTL mapping studies have identified 33 genetic regions on 18 chromosomes with QTL for partial resistance to *P. sojae* [33, 39–50, 52, 56, 61]. Of the 33 regions, four are coincident with *Rps*-genes [9, 10, 12, 15, 63] five are coincident with QTL for resistance to Sudden Death Syndrome [89–93], root disease caused by members of the fungus *Fusarium viguliforme*, 11 are coincident with QTL/genes for resistance to Soybean Cyst Nematode [94–103], and 11 are coincident with QTL for resistance to the necrotrophic fungal pathogen *S. sclerotiorum* causing Sclerotinia stem rot [59, 62, 104, 105]. The coincidence of QTL for partial resistance to *P. sojae* with QTL and *R*-genes for resistance to pathogens with varied lifestyles provides evidence that partial resistance is likely conferred through a variety of different mechanisms.

## Conclusions

In the present study, a GWA analysis was performed to detect genomic regions contributing to quantitative resistance to *P. sojae* using PIs from the Republic of Korea. In addition to identifying five novel QTL, QTLs that coincide with previously reported QTLs for resistance to *P. sojae* were identified. Candidate genes and coincident QTL were identified to explore mechanistic hypotheses of partial resistance, providing evidence towards a number of different hypotheses including a weakened *R-*gene response and genes involved in morphology and development, basal defense and signal transduction. To fully characterize the genes conferring resistance in these regions, functional analyses of candidate genes is necessary or in process.

## Methods

### Seed material

A collection of 1345 PIs from S. Korea were obtained from the National Plant Germplasm System, consisting of 50 seeds per inbred line ranging from maturity groups I to IV. In addition to these lines, checks (‘Conrad’, ‘Sloan’, ‘OX20-8’, ‘Williams 79’, ‘Resnik’, ‘L83-570’, PI 398841 and PI 407861A) with known and varied levels of resistance to *P. sojae* were included in the experimental design. All seeds were vapor phase sterilized following a chlorine gas protocol adapted from Olhoft et al. [106] prior to disease assays.

### Disease assays

Pathogenicity tests were conducted prior to the phenotypic disease assay for selection of two *P. sojae* isolates based on virulence and aggressiveness. Twenty-seven isolates were tested on 15 soybean differentials in a hypocotyl assay for *Rps*-mediated resistance [38] (Additional file 1: Table S1). Virulence was measured by the percentage of susceptible (>90 % compatible reaction), and resistant (<10 % incompatible reaction) responses in 10 soybean seedlings of each differential. Using a growth chamber based tray test [39], the aggressiveness of each isolate was measured according to the mean lesion length of soybean taproots in ten inoculated seedlings from each cultivar, ‘Sloan’ and ‘Conrad’, possessing moderate susceptibility and a high level of partial resistance, respectively [33]. Isolates *OH121* (*vir 1a*, *1b*, *1c*, *1d*, *1k*, *2*, *3a*, *3c*, *4*, *5*, *6*, *7* and *8*) and *C2S1* (*vir 1a*, *1b*, *1c, 1d*, *1k*, *2*, *3a*, *3b*, *3c*, *4*, *5*, *6*, *7* and *8*) were both recovered from soybean in Ohio and were identified as moderately aggressive such that adequate separation of accession with low and high levels of resistance would be expected. OH121 and C2S1 also possesses complex pathotypes (Additional file 1: Table S1) for eliminating *Rps*-mediated responses enabling partial resistance in the greenhouse based layer test [107] could be observed. In order to further assess the potential of *Rps*-mediated response, hypocotyl assays were conducted. Due to the limited seed quantities available for each accession, hypocotyl assays were conducted for only a single isolate (C2S1) and for 94 randomly selected lines which were included in the GWA analysis (Additional file 1: Table S2).

Phenotypic disease assays were conducted on 1398 PIs originating from S. Korea following a layer test protocol adapted from Dorrance et al. [108] to evaluate partial resistance to *P. sojae.* IPH and NPH were averaged from three seedlings per cup. IRW, NRW, ISW, NSW, IRRS and NRRS were measured two weeks after planting. IRRS and NRRS were rated on the 1–9 scale according to Dorrance et al. [107]. Fresh weights of the roots or shoots were adjusted by dividing by the total number of plants per cup to calculate IRW, NRW, ISW and NSW. The full experiment was repeated twice for each isolate. The 1395 PIs were first evaluated with *C2S1*. Lack of root rot is a characteristic of immunity imparted by *Rps*-mediated resistance. Isolates OH121 possesses a pathotype capable of detecting (incompatible, avirulent reaction) *Rps3b* and both isolates may detect novel *Rps* genes. Therefore, in order to limit genetic associations to those involved in partial resistance, PIs that exhibited little to no root rot (mean IRRS ≤ 1.5) were removed from the subsequent disease assays with *OH121*. PIs exhibiting limited root rot (mean IRRS ≤ 1.5) with *OH121* were also removed from the dataset. In addition, PIs with NRRS ≥ 1.5 were removed from the dataset, eliminating possible disease due to contamination or seed-borne pathogens. A total of 800 PIs with a mean IRRS > 1.5 for both isolates and a mean NRRS ≤ 1.5 were further analyzed for heritability and GWA analysis.

### Phenotypic data analysis

BLUP values were generated for each PI for IRRS, IRW, ISW, IPH, NRW, NSW and NPH using PROC MIXED procedure in the software SAS (SAS 9.3, SAS Institute 163 Inc., Cary, NC, USA). Data were analyzed with two models. Using observations from both isolates, the model included interactions with isolate: *Yhjklm* = *μ* + *Ih* + *R*(*I*)*hj* + *K*(*IR*)*hjk* + *Cl + G*(*C*)*lm* + *IG*(*C*)*hlm* + *IChl* + *εhjklm*, where *μ* is the overall mean, *Ih* is effect of the *h*th isolate, *R*(*I*)*hj* is the effect of the *j*th experimental replicate for the *h*th isolate, *K*(*IR*)*hjk* is the effect of the *k*th bench in the *j*th experimental replicate for the *h*th isolate, *Cl* is the effect of the *l*th class of entry [1–9 for Conrad (rps, high levels of partial resistance), L83-570 (*Rps3a*, moderate levels of partial resistance), OX20-8 (*Rps1a*, highly susceptible), PI 398841 (high levels of partial resistance), PI 407861A (high levels of partial resistance), Resnik (*Rps1k*, moderate levels of partial resistance), Sloan (*rps*, moderately susceptible), Williams 79 (*Rps1c*, moderate levels of partial resistance), and experimental lines, respectively], *G*(*C*)*lm* is the effect of the *m*th genotype within class for experimental lines (genotypic variance, σ_g_^2^), *IG*(*C*)*hlm* is the effect of the *h*th isolate with the *m*th genotype within the *l*th class for the experimental lines (genotypic x isolate variance, *σgi*2), *ICh*l is the effect of the *h*th isolate with the *I*th class entry, *εhjklm* is the experimental error (*σε*2). Broad-sense heritability (*H*^2^) was calculated for each trait as follows: *H*^2^ = σ_g_^2^/(σ_g_^2^ + σ_gi_^2^/i + σ_*ε*_^2^/*ir*), where σ_g_^2^ represents the genetic variance, σ_gi_^2^ represents the genotype × isolate variance, σ_*ε*_^2^ represents the error variance, *r* is the number of experimental replicates, and *i* is the number of isolates.

### Genotypic data analysis

Genotypic data [109] collected using the Illumina Infinium SoySNP50K iSelect BeadChip developed by the Beltsville, MD, USDA Soybean Genomics and Improvement Lab were downloaded from Soybase [61]. The genotypic data consisted of 42,509 SNPs [51]. Monomorphic markers and markers with > 5 % missing data or >10 % missing plus heterozygous allele calls were removed. A total of 19,303 polymorphic markers, including 11,126 with minor allele frequencies (MAF) ≥ 5 %, were included in the association analyses. Missing genotypes were imputed with fastPHASE [108]. A genome-wide estimation of LD decay in euchromatic and heterochromatic regions was plotted as physical distance (kbp) vs *r*^*2*^. Population structure was examined using the software ADMIXTURE with five-fold cross-validation [53].

### GWA analyses

GWA analyses of the phenotypic and genotypic data sets for the 797 non-immune cultivars were conducted using the GAPIT (Genome Association Prediction Tool) package [110] in R software [111] with a compressed mixed linear model and population parameters previously determined [112]. The optimal number of principle components for inclusion in the model was determined with GAPIT by Bayesian Information Criterion. The significance threshold for marker-trait associations was determined by a modified Bonferroni adjustment in which M_eff_ was calculated using simpleM [113] Genome-wide threshold levels for each of the two datasets were determined by α/M_eff_, where α = 0.05.

Haplotype blocks were constructed based on the following criteria: 1) markers in significant LD (four-gamete method) [114]; were grouped into haplotype blocks, 2) adjacent blocks separated by < 10 kb were combined. However, no haplotype blocks met criteria 2, thus, no haplotype blocks were combined. For this study, a significant QTL was defined as a haplotype block possessing marker(s) identified through GWA analysis to be significantly associated with a trait. The haplotype block determination and visualization were carried out with Haploview Version 4.2 [115].

## Abbreviations

BLUP, best-linear unbiased predictor; CMLM, compressed mixed linear model; GAPIT, Genome Association Prediction Tool; GWAS, genome-wide association study; IPH, inoculated plant height; IRRS, inoculated root rot score; IRW, inoculated root weight; ISW, inoculated shoot weight; JA, Jasmonic acid; kb, kilobase; LD, linkage disequilibrium; LRR-RLK, leucine rich repeat-receptor like kinase; MAPK mitogen activated protein kinase; M_eff_, effective number of markers; NB-LRR, nucleotide binding-leucine rich repeat; NPH, non-inoculated plant height; NRW, non-inoculated root weight; NSW, non-inoculated shoot weight; PCA, principle component analysis; PI, plant introduction; QTL, quantitative trait loci; RILs, recombinant inbred line; SNPs, single nucleotide polymorphisms

## Additional files

Additional file 1: Table S1. Summary of mean lesion lengths from tray tests and virulence profiles from hypocotyl inoculations utilizing 22 different isolates of *P. sojae*. **Table S2a** Hypocotyl assay with isolate C2S1 and genotypic data of markers within QTL for 94 randomly selected accessions included in the GWA analysis. **Table S2b** Hypocotyl assay of differentials for isolate C2S1. **Figure S1** Examination of population structure of 800 Plant Introductions. **Figure S2** Manhattan plot of the soybean genome depicting the extent of associations of 19,138 SNPs with inoculated root rot score, inoculated root weight, inoculated shoot weight and inoculated plant height. **Figure S3** Manhattan plot of the soybean genome depicting the extent of associations of 19,138 SNPs with non-inoculated root weight, non-inoculated shoot weight and non-inoculated plant height. (PDF 962 kb) Additional file 2: Table S3. Annotations for candidate genes within QTLs. (XLSX 44 kb)
