# Supplementary material for: Genome-wide association mapping of partial resistance to Phytophthora sojae in soybean plant introductions from the Republic of Korea
Source: BMC Genomics. 2016 Aug 11;17:607. doi: 10.1186/s12864-016-2918-5 (PMC4982113; doi:10.1186/s12864-016-2918-5)
Supplement: Additional file 1: Table S1. — Summary of mean lesion lengths from tray tests and virulence profiles from hypocotyl inoculations utilizing 22 different isolates of P. sojae. Table S2a Hypocotyl assay with isolate C2S1 and genotypic data of markers within QTL for 94 randomly selected accessions included in the GWA analysis. Table S2b Hypocotyl assay of differentials for isolate C2S1. Figure S1 Examination of population structure of 800 Plant Introductions. Figure S2 Manhattan plot of the soybean genome depicting the extent of associations of 19,138 SNPs with inoculated root rot score, inoculated root weight, inoculated shoot weight and inoculated plant height. Figure S3 Manhattan plot of the soybean genome depicting the extent of associations of 19,138 SNPs with non-inoculated root weight, non-inoculated shoot weight and non-inoculated plant height. (PDF 962 kb) [file 12864_2016_2918_MOESM1_ESM.pdf]

# Genome-wide association mapping of partial resistance to *Phytophthora sojae* in soybean plant introductions from the Republic of Korea

Rhiannon Schneider[1, 2], William Rolling[3], Qijian Song[4], Perry Cregan[4], Anne E. Dorrance[3, 5], Leah K. McHale[1, 3]

*Corresponding author: Leah K. McHale, mchale.21@osu.edu

[1] Department of Horticulture and Crop Science, The Ohio State University, Columbus, OH, 43210

[2] Current Address: Pioneer Hi-Bred International Inc., Napoleon, OH, 43545

[3] Center for Applied Plant Sciences, The Ohio State University, Columbus, OH 43210

[4] Soybean Genomics and Improvement Laboratory, US Department of Agriculture, Agricultural Research Service, Beltsville, MD 20705

[5] Department of Plant Pathology, The Ohio State University, Wooster, OH 44691

**Supplemental Tables (S1-S2) and Supplemental Figures (S1-S3).**

**Supplemental Table S1.** Summary of mean lesion lengths from tray tests and virulence profiles from hypocotyl inoculations utilizing 22 different isolates of *P. sojae*.

| Isolate | Average Lesion Length (mm) | | Virulence profile |
|---|---|---|---|
| | Conrad | Sloan | |
| 1S11 | 14.19 | 16.56 | *1a, 1b, 1d, 1k, 2, 3a, 3b, 3c, 4, 5, 6, 7, 8* |
| 9d | ND[b] | ND | *1a, 1b, 1c, 1d, 1k, 3a, 3b, 4, 5, 6, 7, 8* |
| Ash 1-1 | 12.44 | 12.08 | *1a, 1b, 1c, 1d, 1k, 3a, 3b, 7, 8* |
| Butler MU S.1. | 9.13 | 15.25 | *1a, 1b, 1c, 1d, 1k, 3a, 7* |
| **C2S1**[a] | **12.50** | **ND** | ***1a, 1b, 1c, 1d, 1k, 2, 3a, 3b, 3c, 4, 5, 6, 7, 8*** |
| DFL-1 | 5.94 | 20.19 | *1a, 1b, 1d, 1k, 7* |
| DP5 | 16.44 | 18.63 | *1a, 1b, 1d, 1k, 3a, 7, 8* |
| MMI12004_04_0.2 | 18.19 | 29.94 | *1a, 1b, 1c, 1d, 1k, 2, 3a, 3b, 3c, 4, 5, 6, 7* |
| OH1200110.1 | 15.88 | 20.69 | *1a, 1b, 1c, 3a, 3b, 5, 6, 7, 8* |
| OH120049.1 | 23.38 | 16.88 | *1a, 1c, 1d, 1k, 2, 3a, 3b, 3c, 5, 7, 8* |
| OH120484.1 | 15.78 | ND | *1a, 1b, 1c, 1d, 1k, 2, 3a, 3b, 5, 7, 8* |
| OH121033.4 | 15.38 | 21.94 | *1a, 1b, 1c, 1d, 1k, 2, 3a, 3c, 4, 7, 8* |
| OH121047.1 | ND | ND | *1a, 1b, 1c, 1d, 1k, 2, 3a, 3b, 3c, 5, 6, 7, 8* |
| OH121074.4 | 10.00 | ND | *1a, 1b, 1c, 1d, 1k, 3a, 3b, 3c, 4, 5, 7, 8* |
| **OH121086.3** | **14.00** | **23.00** | ***1a, 1b, 1c, 1d, 1k, 2, 3a, 3c, 4, 5, 6, 7, 8*** |
| OH121087.1 | 19.88 | 22.06 | *1a, 1b, 1c, 1d, 1k, 2, 3a, 3b, 3c, 5, 6, 7* |
| OH121108.2 | 16.69 | 22.43 | *1a, 1b, 1c, 1d, 1k, 2, 3a, 3b, 3c, 4, 5, 6, 7, 8* |
| OH121187.2 | 12.00 | 20.63 | *1a, 1b, 1c, 1d, 1k, 2, 3a, 3b, 3c, 4, 5, 6, 7* |
| OH121189.1 | 20.92 | ND | *1a, 1b, 1c, 1d, 1k, 2, 3a, 3b, 3c, 4, 5, 6, 7, 8* |
| OH121215.1 | 25.56 | 21.81 | *1a, 1b, 1c, 1d, 1k, 2, 3a, 3b, 3c, 5, 6, 7, 8* |
| OH121018.1 | 17.31 | 16.13 | *1a, 1b, 1c, 1d, 1k, 2, 3a, 3c, 5, 7, 8* |
| OH121215.1 | 29.88 | 28.26 | *1a, 1b, 1c, 1d, 2, 3a, 3b, 3c, 4, 5, 6, 7, 8* |

[a] Isolates selected for disease assays in this study are indicated in bold.

[b] ND: Not determined

**Supplemental Table S2a.** Hypocotyl assay with isolate C2S1 and genotypic data of markers within QTL for 94 randomly selected accessions included in the GWA analysis.

| | | **Resistant (R) or susceptible (S) allele for significant markers at each QTL** | | | | | | | | | | | | | | | |
|---|---|---|---|---|---|---|---|---|---|---|---|---|---|---|---|---|---|
| **Accession** | **No. seedlings dead/Total no. seedlings[a]** | **ss715585712 (QTL 3-1)** | **ss715585728 (QTL 3-1)** | **ss715586320 (QTL 3-2)** | **ss715586321 (QTL 3-2)** | **ss715586376 (QTL 3-3)** | **ss715615031 (QTL 13-1)** | **ss715635897 (QTL 19-1)** | **ss715635934 (QTL 19-2)** | **ss715636056 (QTL 19-3)** | **ss715636059 (QTL 19-3)** | **ss715636064 (QTL 19-3)** | **ss715636073 (QTL 19-3)** | **ss715636076 (QTL 19-3)** | **ss715636077 (QTL 19-3)** | **ss715636083 (QTL 19-3)** | **ss715636084 (QTL 19-3)** |
| PI 398588 | 6/6 | R | S | R | S | S | S | S | S | S | S | S | R | R | R | R | R |
| PI 398600 | 8/8 | S | R | S | R | R | R | S | S | S | S | S | R | R | R | R | R |
| PI 398601 | 8/8 | S | R | S | R | R | S | S | S | S | S | S | R | R | R | R | R |
| PI 398604 | 5/5 | S | R | S | R | R | S | S | S | S | S | S | R | R | R | R | R |
| PI 398620 | 5/5 | S | R | S | R | R | R | S | S | S | S | S | R | R | R | R | R |
| PI 398624 | 4/4 | S | R | S | R | R | R | S | S | S | S | S | R | R | R | R | R |
| PI 398627 | 4/4 | S | R | S | R | R | R | S | S | S | S | S | R | R | R | R | R |
| PI 398637 | 8/8 | S | R | S | R | R | R | S | S | S | S | S | R | R | R | R | R |
| PI 398638 | 8/8 | S | R | S | R | R | S | S | S | S | S | S | R | R | R | R | R |
| PI 398639 | 7/7 | S | R | S | R | S | R | S | S | S | S | S | R | R | R | R | R |
| PI 398641 | 4/4 | S | R | S | R | R | S | S | S | S | S | S | R | R | R | R | R |
| PI 398642 | 5/5 | S | R | S | R | R | R | S | S | S | S | S | R | R | R | R | R |
| PI 398644 | 6/6 | S | R | S | R | R | R | S | S | S | S | S | R | R | R | R | R |
| PI 398654 | 4/4 | S | R | S | R | R | R | S | S | S | S | S | R | R | R | R | R |
| PI 398655 | 6/6 | S | R | S | R | R | R | S | S | S | S | S | R | R | R | R | R |
| PI 398657 | 5/5 | S | R | S | R | R | S | S | S | S | S | S | R | R | R | R | R |
| PI 398686 | 6/6 | R | S | S | R | R | S | S | S | S | S | S | R | R | R | R | R |
| PI 398696 | 6/6 | S | R | S | R | R | R | S | S | S | S | S | R | R | R | R | R |
| PI 398698 | 4/4 | R | S | R | S | S | R | S | S | S | S | S | R | R | R | R | R |
| PI 398706 | 7/7 | S | R | S | R | R | R | S | S | S | S | S | R | R | R | R | R |
| PI 398708 | 7/7 | R | S | R | S | S | S | S | S | S | S | S | R | R | R | R | R |
| PI 398710 | 9/9 | S | R | S | R | R | S | S | S | S | S | S | R | R | R | R | R |
| PI 398712 | 6/6 | S | R | S | R | R | R | S | S | S | S | S | R | R | R | R | R |
| PI 398727 | 5/5 | S | R | S | R | R | S | S | S | S | S | S | R | R | R | R | R |
| PI 398735 | 5/5 | S | R | S | R | R | R | S | S | S | S | S | R | R | R | R | R |
| PI 398744 | 4/4 | S | R | S | R | R | R | S | S | S | S | S | R | R | R | R | R |
| PI 398750 | 10/10 | R | S | R | S | S | S | S | S | S | S | S | R | R | R | R | R |
| PI 398751 | 6/6 | S | R | S | R | R | S | S | S | S | S | S | R | R | R | R | R |
| PI 398752 | 8/8 | S | R | S | R | R | S | S | S | S | S | S | R | R | R | R | R |
| PI 398753 | 4/4 | S | R | S | R | R | S | S | S | S | S | S | R | R | R | R | R |
| PI 398754 | 6/6 | S | R | S | R | R | R | S | S | S | S | S | R | R | R | R | R |
| PI 398776 | 8/8 | S | R | S | R | R | S | S | S | S | S | S | R | R | R | R | R |
| PI 398788 | 7/7 | S | R | S | R | R | S | S | S | S | S | S | R | R | R | R | R |
| PI 398802 | 5/5 | R | S | R | S | S | S | S | S | S | S | S | R | R | R | R | R |
| PI 398803 | 6/6 | R | R | R | R | R | S | S | S | S | S | S | R | R | R | R | R |
| PI 398806 | 8/8 | S | R | S | R | S | R | S | S | S | S | S | R | R | R | R | R |
| PI 398807 | 4/4 | S | R | S | R | R | R | S | S | S | S | S | R | R | R | R | R |
| PI 398808 | 5/5 | S | R | S | R | R | S | S | S | S | S | S | R | R | R | R | R |
| PI 398813 | 7/7 | S | R | S | R | R | S | S | S | S | S | S | R | R | R | R | R |
| PI 398814 | 5/5 | S | R | S | R | R | S | S | S | S | S | S | R | R | R | R | R |
| PI 398816 | 4/4 | S | R | S | R | R | S | S | S | S | S | S | R | R | R | R | R |
| PI 398820 | 4/4 | S | R | S | R | R | S | S | S | S | S | S | R | R | R | R | R |
| PI 398821 | 9/9 | S | R | S | R | R | R | S | S | S | S | S | R | R | R | R | R |
| PI 398822 | 8/8 | S | R | S | R | R | R | S | S | S | S | S | R | R | R | R | R |
| PI 398831 | 8/8 | S | R | S | R | R | R | S | S | S | S | S | R | R | R | R | R |
| PI 398832 | 5/5 | S | R | S | R | R | S | S | S | S | S | S | R | R | R | R | R |

| | | | | | | | | | | | | | | | | | |
|---|---|---|---|---|---|---|---|---|---|---|---|---|---|---|---|---|---|
| PI 398843 | 9/9 | R | S | S | R | S | S | S | S | S | S | S | R | R | R | R | R |
| PI 398858 | 5/5 | R | S | R | S | S | R | S | S | S | S | S | R | R | R | R | R |
| PI 398868 | 5/5 | S | R | S | R | S | R | S | S | S | S | S | R | R | R | R | R |
| PI 398871 | 4/4 | R | S | R | S | R | S | S | S | S | S | S | R | R | R | R | R |
| PI 398878 | 8/8 | S | R | S | R | R | R | S | S | S | S | S | R | R | R | R | R |
| PI 398881 | 9/9 | S | R | S | R | R | R | S | S | S | S | S | R | R | R | R | R |
| PI 398883 | 5/5 | S | R | S | R | R | S | S | S | S | S | S | R | R | R | R | R |
| PI 398885 | 6/6 | S | R | S | R | R | S | S | S | S | S | S | R | R | R | R | R |
| PI 398886 | 5/5 | R | S | R | S | S | S | S | S | S | S | S | R | R | R | R | R |
| PI 398887 | 7/7 | R | S | R | S | S | S | S | S | S | S | S | R | R | R | R | R |
| PI 398888 | 6/6 | S | R | S | R | R | S | S | S | S | S | S | R | R | R | R | R |
| PI 398889 | 6/6, 7/7 | S | R | S | R | R | S | S | S | S | S | S | R | R | R | R | R |
| PI 398891 | 6/6 | S | R | S | R | R | S | S | S | S | S | S | R | R | R | R | R |
| PI 398898 | 8/8 | S | R | S | R | R | R | S | S | S | S | S | R | R | R | R | R |
| PI 398899 | 8/8 | S | R | S | R | R | R | S | S | S | S | S | R | R | R | R | R |
| PI 398901 | 4/4 | S | R | S | R | R | S | S | S | S | S | S | R | R | R | R | R |
| PI 398908 | 7/7 | S | R | S | R | R | S | S | S | S | S | S | R | R | R | R | R |
| PI 398921 | 8/8 | S | R | S | R | R | R | S | S | S | S | S | R | R | R | R | R |
| PI 398923 | 7/7 | R | S | S | R | S | R | S | S | S | S | S | R | R | R | R | R |
| PI 398924 | 10/10 | S | R | S | R | R | S | S | S | S | S | S | R | R | R | R | R |
| PI 398927 | 4/4 | S | R | S | R | R | R | S | S | S | S | S | R | R | R | R | R |
| PI 398929 | 5/5 | S | R | S | R | R | R | S | S | S | S | S | R | R | R | R | R |
| PI 398930 | 8/8 | S | R | S | R | R | S | S | S | S | S | S | R | R | R | R | R |
| PI 398931 | 6/6 | S | R | S | R | R | S | S | S | S | S | S | R | R | R | R | R |
| PI 398934 | 10/10 | S | R | S | R | R | S | S | S | S | S | S | R | R | R | R | R |
| PI 398936 | 5/5 | R | S | S | R | S | R | S | S | S | S | S | R | R | R | R | R |
| PI 398941 | 7/7 | S | R | S | R | R | S | S | S | S | S | S | R | R | R | R | R |
| PI 398942 | 5/5 | S | R | S | R | R | R | S | S | S | S | S | R | R | R | R | R |
| PI 398953 | 10/10 | S | R | S | R | R | R | S | S | S | S | S | R | R | R | R | R |
| PI 398957 | 5/5 | S | R | S | R | S | S | S | S | S | S | S | R | R | R | R | R |
| PI 398960 | 6/6 | S | R | S | R | R | R | S | S | S | S | S | R | R | R | R | R |
| PI 398980 | 6/6 | S | R | S | R | R | R | S | S | S | S | S | R | R | R | R | R |
| PI 398982 | 6/6 | S | R | R | S | S | S | S | S | S | S | S | R | R | R | R | R |
| PI 398987 | 4/4 | S | R | S | R | R | S | S | S | S | S | S | R | R | R | R | R |
| PI 398990 | 5/5 | R | S | R | S | S | R | S | S | S | S | S | R | R | R | R | R |
| PI 398995 | 8/8 | S | R | S | R | R | R | S | S | S | S | S | R | R | R | R | R |
| PI 399010 | 6/6 | S | R | S | R | R | R | S | S | S | S | S | R | R | R | R | R |
| PI 399011 | 7/7 | S | R | S | R | R | S | S | S | S | S | S | R | R | R | R | R |
| PI 399018 | 6/6 | S | R | S | R | R | R | S | S | S | S | S | R | R | R | R | R |
| PI 399028 | 5/5 | S | R | S | R | R | S | S | S | S | S | S | R | R | R | R | R |
| PI 399031 | 4/4 | S | R | S | R | R | R | S | S | S | S | S | R | R | R | R | R |
| PI 399034 | 6/6 | S | R | S | R | R | S | S | S | S | S | S | R | R | R | R | R |
| PI 399035 | 8/8 | S | R | S | R | R | R | S | S | S | S | S | R | R | R | R | R |
| PI 399039 | 7/7 | S | R | S | R | R | S | S | S | S | S | S | R | R | R | R | R |
| PI 399043 | 5/5 | S | R | S | R | R | R | S | S | S | S | S | R | R | R | R | R |
| PI 399046 | 7/7 | S | R | S | R | R | S | S | S | S | S | S | R | R | R | R | R |
| PI 399051 | 7/7 | S | R | S | R | R | S | S | S | S | S | S | R | R | R | R | R |
| PI 399063 | 4/4 | S | R | S | R | R | S | S | S | S | S | S | R | R | R | R | R |

[a] Greater than 75% survival is considered an incompatible reaction, indicating avirulence of the isolate.

**Supplemental Table S2b.** Hypocotyl assay of differentials for isolate C2S1.

| | No. seedlings dead/ Total no. seedlings[a] | | | |
|---|---|---|---|---|
| **Differential line[b]** | **Rep 1** | **Rep 2** | **Rep 3** | **Rep 4** |
| Williams | 12/12 | 8/8 | 7/7 | 9/9 |
| Harlon | 10/10 | 10/10 | 8/8 | 8/8 |
| Harosoy 13XX | 10/10 | 11/11 | 9/9 | 7/7 |
| Williams 79 | 9/9 | 7/7 | 8/8 | 7/7 |
| PI 103091 | 10/10 | 7/7 | 6/7 | 9/9 |
| Williams 82 | 8/8 | 10/10 | 8/8 | 6/6 |
| L76-1988 | 9/9 | 12/12 | 10/10 | 8/8 |
| L83-570 | 10/10 | 9/9 | 11/11 | 11/11 |
| PRX 146-36 | 7/7 | 8/8 | 2/10 | 9/9 |
| PRX 145-48 | 11/11 | 8/8 | 12/12 | 9/9 |
| L85-2352 | 7/7 | 7/7 | 10/11 | 5/5 |
| L85-3059 | 11/11 | 10/10 | 10/10 | 8/8 |
| Harosoy 62XX | 7/7 | 11/11 | 10/10 | 9/9 |
| Harosoy | 12/12 | 9/9 | 11/11 | 8/8 |
| PI 399073 | 5/5 | 6/6 | 4/5 | 5/5 |

[a] Greater than 75% survival is considered an incompatible reaction, indicating avirulence of the isolate.

[b] The Rps genotype of the soybean differentials used to verify the pathotype of C2S1 were as follows: Williams (*rps*/*rps*), Harlon (*Rps1a*/*Rps1a*), Harosoy 13XX (*Rps1b*/*Rps1b*), Williams 79 (*Rps1c*/*Rps1c*), PI 103091 (*Rps1d*/*Rps1d*), Williams 82 (*Rps1k*/*Rps1k*), L76-1988 (*Rps2*/*Rps2*), L83-570 (*Rps3a*/*Rps3a*), PRX 146-36 (*Rps3b*/*Rps3b*), PRX 145-48 (*Rps3c*/*Rps3c*), L85-2352 (*Rps4*/*Rps4*), L85-3059 (*Rps5*/*Rps5*), Harosoy 62XX (*Rps6*/*Rps6*), Harosoy (*Rps7*/*Rps7*), and PI 399073 (*Rps8*/*Rps8*).

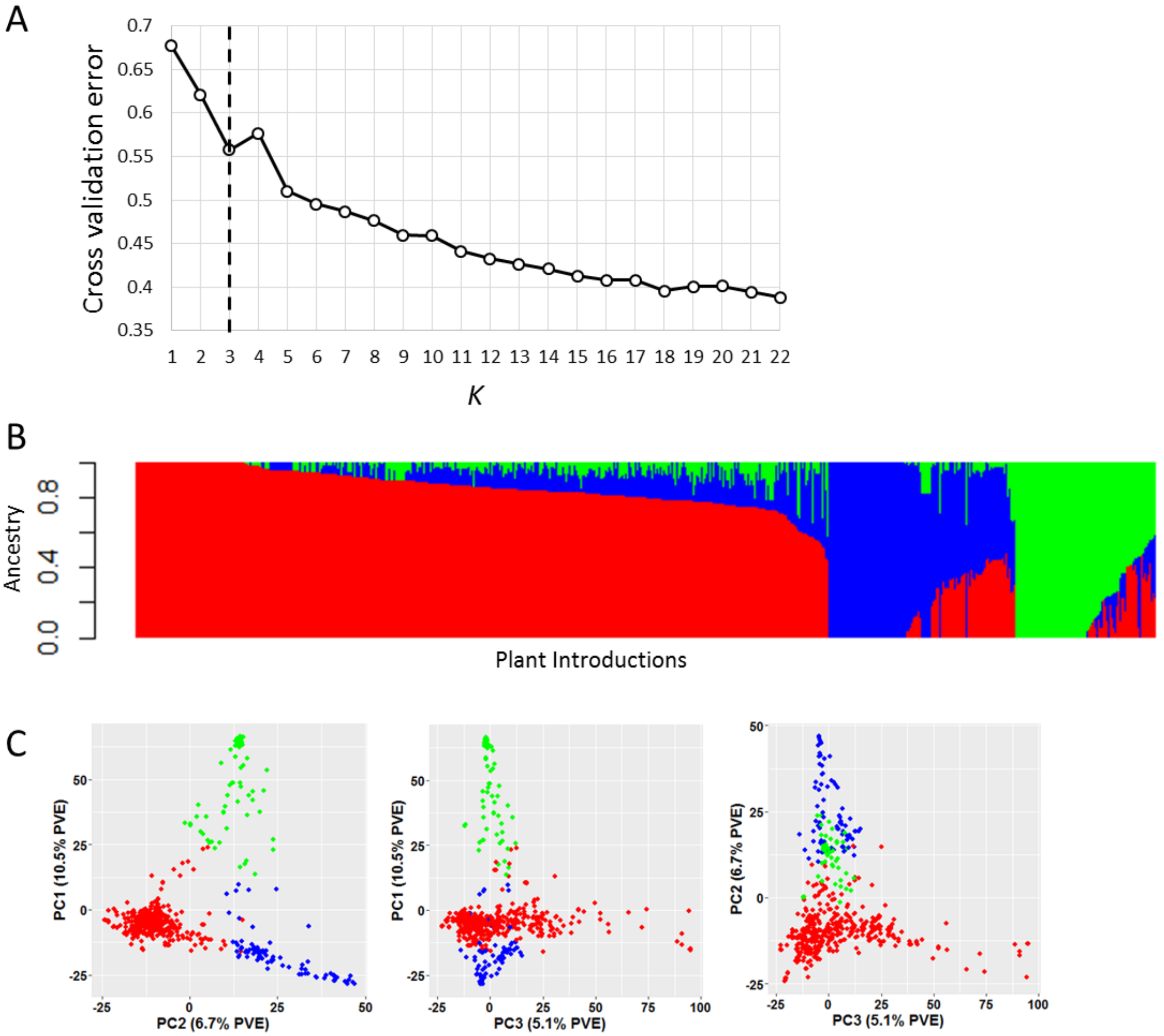


**Supplemental Figure S1.** Examination of population structure of 800 Plant Introductions. Cross validation cross validation error from analysis with ADMIXTURE [1] at K = 1 to K = 22, where the vertical dashed line corresponds with the selected value of K (A). Ancestry of plant introductions based on at K=3 (B). Dispersion of predicted populations at K=3 based on the first three principle components (PCs) with percent variance explained (PVE) for each PC indicated in parentheses (C).

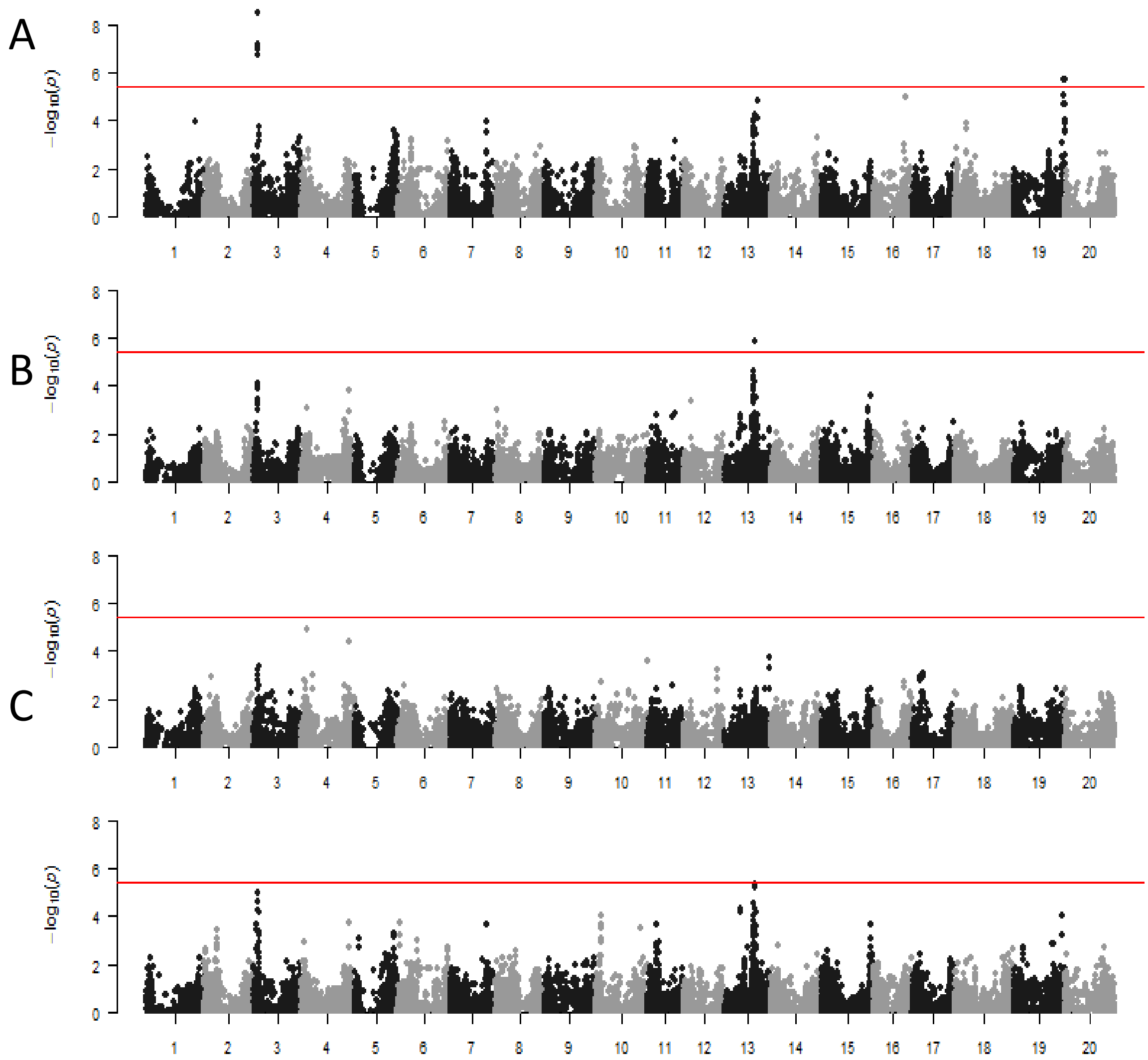


**Supplemental Figure S2.** Manhattan plot of the soybean genome depicting the extent of associations of 19,138 SNPs with inoculated root rot score (A), inoculated root weight (B), inoculated shoot weight (C), and inoculated plant height (D). The $-\log_{10}$ (*p*-value) on the y-axis is a measure of the degree to which a SNP is associated with the trait. Chromosomes are displayed on the x-axis. The red horizontal line indicates the genome-wide threshold for significance.

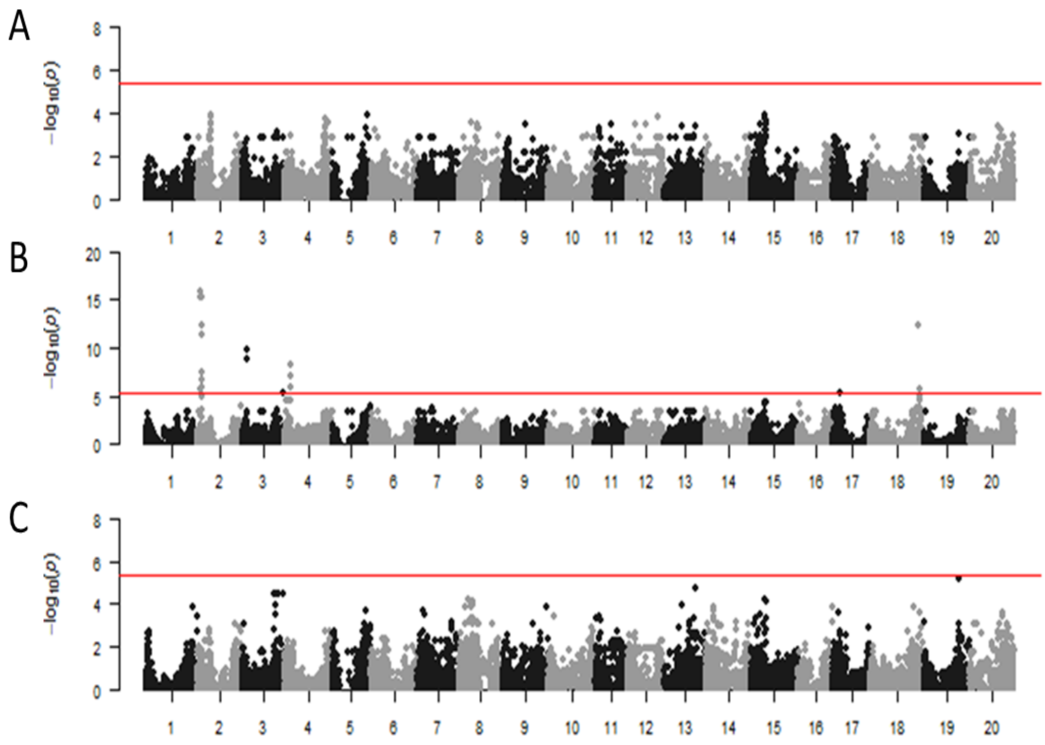


**Supplemental Figure S3**. Manhattan plot of the soybean genome depicting the extent of associations of 19,138 SNPs with non-inoculated root weight (A), non-inoculated shoot weight (B), and non-inoculated plant height (C). The –$\log_{10}$(p-value) on the y-axis is a measure of the degree to which a SNP is associated with the trait. Chromosomes are displayed on the x-axis.  The red horizontal line indicates the genome-wide threshold for significance.

## References


1. Alexander DH, Novembre J, Lange K. Fast model-based estimation of ancestry in unrelated individuals. Genome Res. 2009, 19:1655–1664.
